# The Development of a Model to Predict Sports Participation among College Students in Central China

**DOI:** 10.3390/ijerph19031806

**Published:** 2022-02-05

**Authors:** Tianzhi Liao, Saizhao Tang, Yunsik Shim

**Affiliations:** 1 Department of Sports Science, Soonchunhyang University, 22 Soonchunhyangro, Asan 31538, Korea; yocheomji@gmail.com; 2 Department of Sports Medicine, Soonchunhyang University, 22 Soonchunhyangro, Asan 31538, Korea; tangsaizhao@gmail.com

**Keywords:** competence, relatedness, autonomy, theory of planned behavior, Central China college students

## Abstract

This study applies the theory of planned behavior (TPB) and self-determination theory (SDT) to predict the sports participation and exercise intentions of college students in Central China by considering the mediating roles of attitudes, subjective norms, and perceived behavioral control. Structural equation modeling (SEM) was used to analyze self-reported data from 294 college students (144 males and 150 females). The relationship between the research variables was tested by the mediation model and Bootstrap 5000 sampling using AMOS version 24. The results show that the direct effects of attitudes and perceived behavioral control on motor intention and motor participation are significant in the model. The satisfaction of the three psychological needs had a positive indirect effect on motor participation through attitudes; competence and autonomy had a positive indirect effect on motor participation mediated through subjective norms; however, only competence had a positive indirect effect on motor mediated through perceived behavioral control. In conclusion, this research demonstrates the importance of meeting these three basic psychological needs when designing intervention measures to promote college students’ sports participation.

## 1. Introduction

Long-term regular exercise is good for people’s health [1,2] and can prevent diseases [3], but college students’ participation in physical activities is low. Only 39.6% of college students in China engage in the recommended level of exercise [4], which is much lower than the 49.9% of U.S. college students who regularly engage in moderate to vigorous activity [5]. Moreover, in Healthy Campus 2010, physical inactivity was listed as one of the six health risk behaviors among college students [6]. For young people, the college-age period involves the transition from adolescence to adulthood. During this time, college students develop their lifestyles, and the various habits they acquire will affect both their current and future health [7]. Physical activity patterns established during college are maintained for extended periods [3]. College students are likely to keep these patterns throughout their lifetime, affecting their health over time [8]. Therefore, understanding the influencing factors of college students’ sports participation has become a significant public health priority. Effective sports promotion plans are needed to increase college students’ involvement in physical activities [9]. However, a valid generalization requires reasonable theoretical models [10], and thus it is very important to determine the most effective theoretical model to accomplish this goal [11].

### 1.1. Theory of Planned Behavior

The theory of planned behavior (TPB) is one of the most commonly used models for predicting health-related behaviors [12,13]. The TPB is a theory that links individuals’ beliefs and behaviors. The theory states that intentions regarding attitudes, subjective norms, and perceived behavioral control combine to shape individuals’ behavioral intentions and behaviors. These factors indicate behavioral intentions, but the relative importance of each depends on differences in behavior types and scenarios. Attitudes refer to a subject’s evaluations of specific behaviors (positive/negative evaluations). Subjective norms refer to the social pressures (social approvals/disapprovals) that an individual perceives when deciding whether to perform a specific behavior. Perceived behavioral control reflects whether an individual can control or master particular behaviors (easy/difficult) [14,15,16]. Theoretically, the TPB model suggests that people with positive attitudes who perceive strong support from their significant others and maintain high self-confidence, competence, and control over their physical activities will have a higher willingness to engage in physically active behaviors.

Physical exercise is a deliberate process and planned behavior [17]. When people evaluate a particular behavior positively (attitudes), they intentionally engage in that behavior. When others who perceive it as necessary want an individual to engage in a behavior (subjective norms) and the individual believes that the behavior is under his or her control (perceived behavioral control), this is when solid intentions and perceived behavioral control increase the likelihood that the behavior will occur [13]. Many studies have confirmed that intention is the best predictor of conduct and that physical exercise intention is a close and direct predictor of physical exercise behavior [18]. Sports intention plays a crucial role in sports behavior, so it is essential to understand this intention’s formation [12].

The TPB has been successfully applied to several behavioral domains, and the vast majority of studies have demonstrated its useful explanatory and predictive power [16]. Fishbein and Ajzen proposed that the TPB can accommodate additional predictors to improve its predictive ability [19]. In the study of sports and health, integrating the concept of psychological needs satisfaction from the self-determination theory (SDT) into motivational theories, such as the TPB, can further improve the interpretative and predictive capabilities of such models [20,21,22]. The TPB focuses on explaining differences between intentions and health-related behaviors, but cannot determine the sources of motivations [23]. Thus, the SDT can explain the origin of the TPB’s structure [24], given that the TPB is a supplementary explanation for the processes behind motivational behaviors. The structure and assumptions of these two theories are therefore integrated into a unified motivation model to explain and predict both intentions and health-related behaviors [25,26].

### 1.2. Self-Determination Theory 

SDT involves the psychological needs and personal motivations of humans to engage in certain behavioral activities [27,28,29,30]. SDT emphasizes the degree of self-determination in human behavior, and suggests that the social environment can enhance internal motivation, promote the internalization of external motivation, and ensure healthy human growth by supporting the satisfaction of three basic psychological need for competence, relatedness, and autonomy. Satisfying these three basic psychological needs is essential for happiness and behavioral continuity [31]. The need for competence concerns whether individuals feel valued in their ongoing interactions with their social environments and experience opportunities to express their capabilities. The need for autonomy refers to how individuals think freely and are responsible for their behaviors. Finally, the need for relatedness stems from whether individuals feel a certain sense of belongingness and connectedness to others in their social environments [32].

The integration of the structures and assumptions of both theories into a unified model of motivation explains and predicts both intentions and health-related behaviors [26,33]. Individuals who feel that their psychological needs are met are more likely to be motivated and persistent in their tasks [29]. Motivation plays a crucial role in physical activity intentions and different attitude levels, and subjective norms and perceived behavioral control may arise from other types of motivation [30]. The combination of SDT and the TPB provides additional explanations for processes that cannot be explained by each view alone. The combination of the SDT and the TPB offers complementary bases for the methods that neither approach can explain. That is, the inclusion of SDT helps researchers illustrate the qualities of the TPB [34,35] and its initial conditions [36,37].

This study therefore harnesses these two prominent social psychology theories. It integrates their structures and assumptions into motivation models to explain both intentions and health-related behaviors and to further explore the relationships between these variables. Thus, SDT’s basic needs of competence, relatedness, and autonomy are incorporated into the TPB as background variables to predict physical activity behaviors, promote sports participation, and improve the efficiency of physical activities [22].

### 1.3. Aim of the Present Study

**Figure 1:**
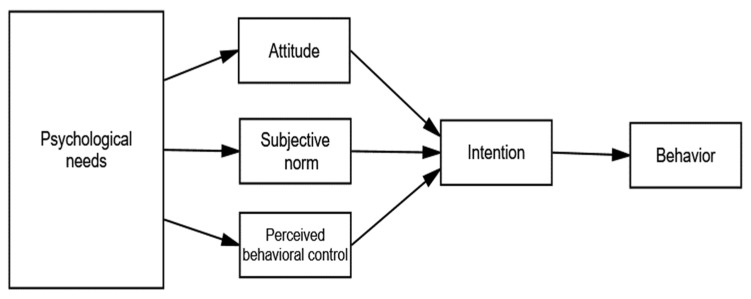
The TPB with the SDT’s variables as background variables.

In this study, as Figure 1 shows, SDT’s three psychological needs were incorporated into the TPB using a structural equation model to determine the difference between the effects of these needs on college student participation in sports. Accordingly, the current study addressed the following two research questions:

1. When integrating SDT and the TPB, how do the basic psychological needs from SDT enhance sports participation and exercise intentions?
2. What intermediary roles do the TPB’s three factors play in the relationship between sports participation and basic psychological needs?

## 2. Methodology

### 2.1. Data

The participants of this study included 311 undergraduate students of Jishou University, which is a large public university located in central China. They were enrolled in a course called ‘Public Physical Education’, one of the core curriculum courses required for all undergraduate students. Although structural equation modeling does not provide an accurate sample size standard, Dawn Iacobucci (2010) recommends a sample size of 200 or more [38]. Thus, the evaluation of the parameters was determined to be very stable, and the significance test had statistical significance.

In total, this study analyzed 294/311 sets of data (94.53%; 17 unreliable responses), which met the recommended sample size and satisfied the minimum number of participants. Table 1 presents the descriptive statistics of the participants. Of 294 students (144 males and 150 females), the mean score of sports participation was 101 min/week. The mean age was 22.08 years. Most participants were freshmen (27.21%), more than half preferred jogging (57.82%), and most exercised more than 3 times a week (55.78%). Each exercise period was more than 20 min (55.45%), and most of the exercise habits lasted more than one month.

### 2.2. Measures

Ajzen’s planned behavior theory includes attitudes, subjective norms, perceived behavioral controls, and intentions [39,40]. Shen revised and compiled the TPB scale in China in 2010 [41]. In this study, the 14 items were scored on a 7-point Likert scale, ranging from very unlikely to very likely. Five questions measure the attitude toward sports participation. Participants reported their attitudes toward participating in sports using the differential semantic measures of ‘Harmful or Beneficial’, ‘Unpleasant or Pleasant’, ‘Good or Bad’, ‘Valuable or Worthless’, and ‘Unenjoyable or Enjoyable’ (Cronbach’s a = 0.960). Subjective norms were measured with three questions, including: ‘Most people who are important to me approve of my exercising for at least 20 min, three times per week for the next four weeks’ (Cronbach’s a = 0.936). To assess perceived behavioral control, participants answered three items, including: ‘I am confident that I can exercise for at least 20 min, three times per week for the next three months’ (Cronbach’s a = 0.950). Sports participation intentions were measured with three questions, such as: ‘I intend to exercise for at least 20 min, three times per week for the next four weeks’ (Cronbach’s a = 0.954). Using AMOS for confirmatory factor analysis (CFA), the final TPB model fit was recorded as CMIN = 251.448, DF = 112, CMIN/DF = 2.245, RMSEA = 0.065, CFI = 0.973.

Deci’s SDT includes the variables of competence, relatedness, and autonomy [42]. Thus, this study used 18 items to measure the satisfaction of the psychological needs of competence (e.g., ‘I feel free to exercise in my way’, Cronbach’s a = 0.936), relatedness (e.g., ‘I feel attached to my exercise companions because they accept me for who I am’, Cronbach’s a = 0.936), and autonomy (e.g., ‘I feel that I am able to complete exercises that are personally challenging’, Cronbach’s a = 0.936). These items were scored on a 7-point Likert scale, ranging from not true at all to very true. Using AMOS for CFA, each of the three items had a single inconsistency that was deleted (COM1, REL6, AUT6). The fit of the final SDT model satisfies CMIN = 240.84, DF = 87, CMIN/DF = 2.768, RMSEA = 0.77, and CFI = 0.967.

Participants were explicitly instructed to provide a rating on the scales according to how they felt during sports participation. The general sociodemographic characteristics of the participants in terms of gender (male or female), age (years), and college year (freshman, sophomore, junior, or senior) were also assessed. Sports participation was measured by asking participants to report the total time (minutes) they spent participating in sports during the week, their sports, their number of exercises per week, the duration of each activity, and their exercise habits.

### 2.3. Procedures

Two English professionals translated these items into Chinese, and two other translators translated them back into the original language. Ultimately, psychology experts (*n* = 2) and physical education experts (*n* = 1) determined that there was no need for further modifications. Thus, after receiving the final version, we collected questionnaires from May to June 2021. After the study participants read the respondents’ informed consent form and fully understood the purpose of the study and anonymity of their responses, the students agreed to voluntarily participate in the exercise behavior survey, and the online questionnaire took approximately 10 min to complete. Each survey was conducted in the presence of a member of the research team.

### 2.4. Statistical Analysis

This research used structural equation modeling (SEM) to test the model. One advantage of SEM is that it simultaneously considers the assessment of the measurement model and the estimation of the structural coefficients. We examined the direct and indirect influences of the following variables on sports participation among Chinese university students: precursor background variables (i.e., the basic psychological needs of competence, relatedness, and autonomy), perceived behavioral controls, subjective norms, and attitudes.

First, outliers and missing values were determined and nonconforming cases were eliminated. Second, IBM SPSS version 23 performed reliability and validity testing and exploratory factor analysis. Next, using AMOS version 24 to perform first-order and higher-order confirmatory factor analysis (CFA), SDT and the TPB obtained satisfactory fitting indices, indicating that the next analysis step could be carried out. Finally, AMOS version 24 was also used to perform SEM. Barrett (2007) generally uses the built-in maximum likelihood method. All estimated parameters were significantly different from 0 at a 95% confidence level (*p* < 0.05) [43]. All variables were continuous, and this study used CFA to examine a measurement model that contains five correlated latent variables (i.e., the satisfaction of the three types of psychological needs, subjective norms, attitudes, perceived behavioral controls, and intentions).

Indirect effects used the bootstrap method. If a sample size is small, the bootstrap test may incorrectly assume the normality of an indirect impact [44,45]. Schumacker and Lomax (2010) used a model fit index to evaluate the goodness-of-fit: approximate value (RMSEA, generally recommended within 0.08 to indicate that a model works well, within 0.1 is acceptable), the ratio of the chi-square to degrees of freedom (CMIN/DF, generally recommended to be between 1 and 3), and the comparative fitting index (CFI, which is recommended to be above 0.90 to indicate that a model fits well) [46].

## 3. Results

### 3.1. Model Fit and Direct Effects

**Figure 2:**
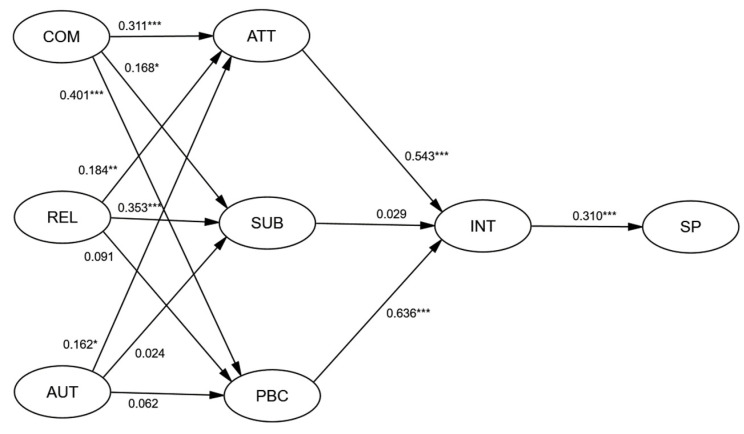
Full research models. Note: * *p* < 0.05, ** *p* < 0.01, *** *p* < 0.001; COM = competence; REL = relatedness; AUT = autonomy; ATT = attitudes; SUB = subjective norms; PBC = perceived behavioral control; INT = intentions; SP = sports participation. All path coefficients have been standardized.

After successfully fitting the CFA, AMOS version 24 was used to conduct a complete model study. The standardized paths from each latent variable to its items were examined. We allowed correlations between all latent variables in the model specification. In our three measurement models, although the chi-square values were all significant (*p* < 0.05). As shown in Figure 2, the full hypothesized structural equation model achieved a good fit to the data observed [46]: CMIN/DF = 2.625, RMSEA = 0.074, CFI = 0.920. As reported in Table 2, the path coefficients were significant and in the right direction for ATT-INT (standardized coefficient β = 0.543), SUB-INT (β = 0.029), and the PBC-INT relationship (β = 0.636). The standardized path coefficient from competency, relatedness, and autonomy to attitudes made a significant contribution to the model (*p* < 0.05). The coefficient from competency and relatedness to subjective norms was significant (*p* < 0.05). The coefficient from competency to perceived behavioral control was significant (*p* < 0.001). The coefficient from attitudes and perceived behavioral control to intentions was significant (*p* < 0.001). The coefficient from intentions to sports participation was significant (*p* < 0.001). The rest of the paths are not significant.

### 3.2. Indirect Effects

Regarding the multiple mediation models, the researcher is concerned with the specific indirect effects of psychological needs on sports participation. These influential specific effects and critical ratios are reported in Table 3; the confidence interval for each indirect effect can be calculated by convention. The indirect effects of additional background variables on sports participation were examined through the three components of the TPB and intentions. The satisfaction of all three psychological needs positively and indirectly affects sports participation through attitudes. Competency and autonomy had positive indirect effects through the subjective norm-mediated regulation of motor participation. However, only competency had a positive indirect effect through perceived behavioral control’s mediation of movement production. Competency, relatedness, and autonomy also positively and indirectly impact sports participation.

## 4. Discussion

Research on the TPB dominates this field [13], and thus, many studies and meta-analyses consistently report the close relationship between the variables of the TPB and physical activities [47,48,49]. According to Noar and Zimmerman’s recommendations, researchers combine the TPB with SDT by leveraging their theoretical advantages for describing health to improve their interpretations and predictions through a holistic model that promotes approach- and reality-based intervention plans [20,22,50]. Accordingly, this study attempts to answer the following questions: (1) When applying the TPB, what are the differences in the direct impacts of the three types of psychological needs on sports participation? (2) How do the three psychological needs indirectly affect sports participation?

This research helps to clarify the influence of the basic psychological needs of SDT on TPB. The results show that (1) according to the TPB model, attitudes toward the three psychological needs for exercise participation were significantly related to intentions, and ability was also related to perceived behavioral control and intentions. However, subjective norms do not affect intentions. Perceived behavioral control is the most effective predictor of intentions. The influence of attitudes on intentions is similar to that of perceived behavioral control. (2) The satisfaction of all three psychological needs positively and indirectly affects sports participation through attitudes. Competency and autonomy had positive indirect effects through the subjective norm-mediated regulation of motor participation. However, only competency mediated a positive indirect effect through perceived behavioral control’s mediation of movement production.

This direct effect research shows that attitudes and behavioral control explain intentions better than subjective norms [51,52]. Subjective norms contributed less to intention prediction than attitudes and perceived behavioral control, with similar forecast levels for attitudes and perceived behavioral control that are consistent with prior research on other contexts [47,53,54]. When the three basic psychological needs were used as background variables, and attitudes, subjective norms, and perceived behavioral control were used as mediators, different degrees had a positive impact on exercise intention and exercise participation. Only six of the 9 effect sizes were moderated by the proposed moderators. Path analysis results show that the significant effects of self-determined motivation on intentions and behavior were partially mediated by the proximal predictors from the TPB. 

In the TPB, the construction of subjective norms reflects a form of social influence pressure. Subjective norms measure the perceptions of pressures from others [13], so the direct link between subjective norms and intentions increases as perceived social pressures increase. Perceived social pressure is a hindrance on rather than a facilitator of motivation [29]. When social pressure exceeds a threshold, it can harm attitudes. Thus, subjective norms may not predict intentions. The relationship with intentions is therefore not significant because socially influential pressures that construct subjective norms do not necessarily promote intentions [55]. The insignificance of subjective norms may also stem from research on the TPB that supports group norms and group identifications. That is, individual behaviors tend to be influenced by group attitudes and intentions to the extent that an individual identifies with a group. When individuals do not identify with a group, group process behavior is not a strong determinant; attitudes, perceived behavioral control, and intentions significantly influence individual behavior variables [56,57]. Although the TPB assumes that subjective norms help explain behavioral intentions, the social pressures and group identity implicit in this result may inhibit the realization of the relevant behavior.

Essential psychological satisfaction generates exercise behaviors through different processes, suggesting that integrating SDT into the TPB may more accurately explain the effect of social factors on this variable. Engaging in physical activity is purposeful and intentional [12]. Therefore, it is essential to understand the motivational processes involved in such voluntary behavior [13]. The integration of the TPB and SDT provides a complementary explanation for the motivational processes involved in planned behaviors [22,35]. Notably, the relationships in the models were all relatively stable, reinforcing the idea that essential psychological satisfaction may play a relevant and crucial role in explaining exercise behaviors. Therefore, the model clearly articulates the differences in physical activity intentions, providing a theoretical basis for further analyses using this integrative model in physical activity-related studies.

Despite the limitations of the research, the results of this study are still valuable. Although it has demonstrated the critical use of SDT’s psychological needs as a background variable in planned behavior theory to predict sports participation, the study has the following limitations: First, it uses a cross-sectional design that provides past or present behavioral measurements rather than estimations of future behaviors. Because of its limitations, there may be consistency deviations, which makes it impossible to determine the causal relationships between variables. Second, the use of a self-reporting instrument is a limitation of this study. Such self-reporting will cause variations in the independent variables, as will the human factors between the variables, due to the evaluator, measurement context, and contents and characteristics of the question items, all of which may cause participants to report differently. Thus, although efforts have been made to control for the ambiguity of the question items, the format of the scale, and the length of the questionnaire, some uncontrollable factors in the research, such as social expectations and subjects’ response emotions, may still affect the results of the investigation. Finally, the object of this study was college students in the same school in Central China, so it is necessary to be cautious about generalizing its results to other populations. This research is exploratory. Future research should first conduct a small sample survey. After the survey results are confirmed, other scholars should then conduct similar studies. This research model should be applied and extended to different groups. Thus, the sample size will continue to increase, and integrated future research can also be performed. 

## 5. Conclusions

In conclusion, the present study affirms applying an integrated theoretical model consisting of the TPB and SDT in physical activity research. Our findings corroborate evidence from previous studies that highlight the role of SDT and the TPB in predicting volitional behavior and physical activity. Studies guided by theory promote an understanding of adherence to health behaviors among researchers and research subjects. Therefore, the combination of planned behavior and SDT provides a valuable framework for explaining health behaviors.

This study helps elucidate the role of SDT’s social influences within the TPB. The results suggest that SDT’s social factors may play a more relevant role in explaining exercise intentions in college than the TPB’s social factors. The basic psychological needs of the individual have always been a social factor positively associated with exercise behaviors. The relationship between attitudes and perceived behavioral controls and exercise intentions is relatively strong, while that between subjective norms and exercise intentions is relatively weak. Therefore, the social factors that cause an individual to feel that there is no pressure on his or her behavioral purpose or performance may be more conducive to that individual’s exercise participation. However, those factors involving different forms of social anxiety with varying degrees of group identity may prevent individuals from engaging in future behaviors. Finally, our results show that creating a social environment of satisfaction, autonomy, and relevance is very important to encourage sports participation among college students. This study confirms that psychological needs contribute to the interpretation of intentions by the TBP model. At the same time, meeting psychological needs when sports intervention is involved can help improve college students’ sports participation.

## Figures and Tables

**Table 1:** Sociodemographic characteristics of participants (*n* = 294).

Characteristics		Number (Persons)	Percent (%)
Gender	Males	144	49.00
	Females	150	51.00
Age	18	8	2.72
	19	13	4.42
	20	45	15.31
	21	42	14.29
	22	59	20.07
	23	63	21.43
	24	33	11.22
	25	27	9.18
	26	3	1.02
	27	1	0.34
College year	Freshman	80	27.21
	Sophomore	62	21.09
	Junior	77	26.19
	Senior	75	25.51
Sport	Jogging	170	57.82
	Team sport	34	11.57
	Dancing	35	11.91
	Aerobic activity	17	5.78
	Walking	10	3.40
	Other	28	9.52
Per week	No	6	2.04
	1–2 times	124	42.18
	3–4 times	93	31.63
	5–6 times	50	17.01
	7 times	21	7.14
Time per exercise	Less than 10 min	20	6.80
	11–20 min	111	37.75
	21–30 min	71	24.15
	31–60 min	54	18.37
	60 min or more	38	12.93
Habits	Less than 1 months	64	21.77
	1–3 months	130	44.22
	3–6 months	46	15.65
	6 months–1 year	21	7.14
	More than 1 year	33	11.22

**Table 2:** Standardized parameter estimates for the research model.

Path (Direct Effects)	Parameter				
	Estimate	S.E.	C.R.	*p*-Value	Std.
COM → ATT	0.313	0.07	4.476	***	0.311
COM → SUB	0.146	0.063	2.299	0.022	0.168
COM → PBC	0.397	0.072	5.495	***	0.401
AUT → ATT	0.203	0.091	2.238	0.025	0.162
AUT → SUB	0.026	0.082	0.315	0.753	0.024
REL → SUB	0.357	0.076	4.701	***	0.353
AUT → PBC	0.077	0.093	0.827	0.408	0.062
REL → PBC	0.104	0.083	1.263	0.207	0.091
REL → ATT	0.215	0.08	2.678	0.007	0.184
ATT → INT	0.295	0.047	6.301	***	0.543
SUB → INT	0.018	0.05	0.362	0.718	0.029
PBC → INT	0.352	0.052	6.789	***	0.636
INT → SP	0.253	0.063	4.012	***	0.310

Note: *** *p* < 0.001; COM = competence; REL = relatedness; AUT = autonomy; ATT = attitudes; SUB = subjective norms; PBC = perceived behavioral controls; INT = intentions; SP = sports participation. All path coefficients were standardized.

**Table 3:** The indirect effects of the self-determination theory construct on sport participation.

Indirect Effects				Coefficients
Competency	Attitude	Intention	Sport participation	0.022 (***)
	Subjective norm	Intention	Sport participation	0.003 (*)
	Perceived behavioral control	Intention	Sport participation	0.031 (***)
Relatedness	Attitude	Intention	Sport participation	0.014 (*)
	Subjective norm	Intention	Sport participation	0.001
	Perceived behavioral control	Intention	Sport participation	0.006
Autonomy	Attitude	Intention	Sport participation	0.015 (***)
	Subjective norm	Intention	Sport participation	0.007 (*)
	Perceived behavioral control	Intention	Sport participation	0.008

Note: COM = competency; REL = relatedness; AUT = autonomy; ATT = attitudes; SUB = subjective norms; PBC = perceived behavioral controls; INT = intentions; SP = sports participation; BC = bias corrected; 5000 bootstrap samples, * *p* < 0.05, *** *p* < 0.001; * Denotes a statistically significant regression coefficient (i.e., the 95% CI does not contain the zero value).

## Data Availability

Not applicable.

## References

[1] Bouchard C., Shephard R.J., Stephens T.E. (1994). Physical activity, fitness, and health: International proceedings and consensus statement. Proceedings of the International Consensus Symposium on Physical Activity, Fitness, and Health.

[2] U.S. Department of Health and Human Services, Centers for Disease Control and Prevention, National Center for Health Statistics (2000). Healthy People 2010: Understanding and Improving Health.

[3] Fish C., Nies M.A. (1996). Health promotion needs of students in a college environment. Public Health Nurs..

[4] Yi Z. (2015). The Research on the Extracurricular Physical Exercise Current Situation and Countermeasures of College Student in Hubei Province.

[5] Dinger M.K., Brittain D.R., Hutchinson S.R. (2014). Associations between physical activity and health-related factors in a national sample of college students. J. Am. Coll. Health.

[6] Mack D.E., Wilson P.M., Lightheart V., Oster K., Gunnell K.E. (2009). Healthy Campus 2010: Physical activity trends and the role information provision. J. Phys. Act. Health.

[7] Gordon-Larsen P., Nelson M.C., Popkin B.M. (2004). Longitudinal physical activity and sedentary behavior trends: Adolescence to adulthood. Am. J. Prev. Med..

[8] Sparling P.B., Snow T.K. (2002). Physical activity patterns in recent college alumni. Res. Q. Exerc. Sport.

[9] Armitage C.J. (2005). Can the theory of planned behavior predict the maintenance of physical activity?. Health Psychol..

[10] Grol R.P., Bosch M.C., Hulscher M.E., Eccles M.P., Wensing M. (2007). Planning and studying improvement in patient care: The use of theoretical perspectives. Milbank Q..

[11] Glanz K., Rimer B.K., Viswanath K. (2008). Health Behavior and Health Education: Theory, Research, and Practice.

[12] Ajzen I. (1985). From intentions to actions: A theory of planned behavior. Action Control.

[13] Ajzen I. (1991). The theory of planned behavior. Organ. Behav. Hum. Decis. Processes.

[14] Ajzen I. (2001). Nature and operation of attitudes. Annu. Rev. Psychol..

[15] Ajzen I. (2002). Perceived behavioral control, self-efficacy, locus of control, and the theory of planned behavior 1. J. Appl. Soc. Psychol..

[16] Conner M., Sparks P. (2005). Theory of planned behaviour and health behaviour. Predict. Health Behav..

[17] Aarts H., Paulussen T., Schaalma H. (1997). Physical exercise habit: On the conceptualization and formation of habitual health behaviours. Health Educ. Res..

[18] Hagger M.S., Chan D.K.C., Protogerou C., Chatzisarantis N.L.D. (2016). Using meta-analytic path analysis to test theoretical predictions in health behavior: An illustration based on meta-analyses of the theory of planned behavior. Prev. Med..

[19] Fishbein M., Ajzen I. (2011). Predicting and Changing Behavior: The Reasoned Action Approach.

[20] Hagger M.S., Chatzisarantis N.D.L., Harris J. (2006). From psychological need satisfaction to intentional behavior: Testing a motivational sequence in two behavioral contexts. Personal. Soc. Psychol. Bull..

[21] Hagger M.S., Chatzisarantis N.D.L. (2009). Integrating the theory of planned behaviour and self-determination theory in health behaviour: A meta-analysis. Br. J. Health Psychol..

[22] Al-Jubari I., Arif H., Liñán F. (2019). Entrepreneurial intention among University students in Malaysia: Integrating self-determination theory and the theory of planned behavior. Int. Entrep. Manag. J..

[23] Chatzisarantis N.D.L., Hagger M.S., Smith B. (2007). Influences of perceived autonomy support on physical activity within the theory of planned behavior. Eur. J. Soc. Psychol..

[24] Andersen S.M., Chen S., Carter C. (2000). Fundamental human needs: Making social cognition relevant. Psychol. Inq..

[25] Standage M., Duda J.L., Ntoumanis N. (2003). A model of contextual motivation in physical education: Using constructs from self-determination and achievement goal theories to predict physical activity intentions. J. Educ. Psychol..

[26] Wilson P.M., Rodgers W.M. (2004). The relationship between perceived autonomy support, exercise regulations and behavioral intentions in women. Psychol. Sport Exerc..

[27] Deci E.L., Ryan R.M. (1985). The general causality orientations scale: Self-determination in personality. J. Res. Personal..

[28] Deci E.L., Flaste R. (1995). Why We Do What We Do: The Dynamics of Personal Autonomy.

[29] Deci E.L., Ryan R.M. (2000). The what and why of goal pursuits: Human needs and the self-determination of behavior. Psychol. Inq..

[30] Deci E.L., Ryan R.M. (2012). Motivation, personality, and development within embedded social contexts: An overview of self-determination theory. The Oxford Handbook of Human Motivation.

[31] Teixeira P.J., Silva M.N., Mata J., Palmeira A.L., Markland D. (2012). Motivation, self-determination, and long-term weight control. Int. J. Behav. Nutr. Phys. Act..

[32] Ryan R.M., Deci E.L. (2002). Overview of self-determination theory: An organismic dialectical perspective. Handb. Self-Determ. Res..

[33] Wilson P.M., Mack D.E., Grattan K.P. (2008). Understanding motivation for exercise: A self-determination theory perspective. Can. Psychol. Psychol. Can..

[34] Chatzisarantis N.D.L., Biddle S.J.H., Meek G.A. (1997). A self-determination theory approach to the study of intentions and the intention–behaviour relationship in children’s physical activity. Br. J. Health Psychol..

[35] Chatzisarantis N.D.L., Biddle S.J.H. (1998). Functional significance of psychological variables that are included in the Theory of Planned Behaviour: A Self-Determination Theory approach to the study of attitudes, subjective norms, perceptions of control and intentions. Eur. J. Soc. Psychol..

[36] Chatzisarantis N.D.L., Hagger M.S., Biddle S.J.H., Karageorghis C. (2002). The cognitive processes by which perceived locus of causality predicts participation in physical activity. J. Health Psychol..

[37] Hagger M.S., Chatzisarantis N.D.L., Biddle S.J.H. (2002). The influence of autonomous and controlling motives on physical activity intentions within the Theory of Planned Behaviour. Br. J. Health Psychol..

[38] Iacobucci D. (2010). Structural equations modeling: Fit indices, sample size, and advanced topics. J. Consum. Psychol..

[39] Ajzen I. (2006). Constructing a Theory of Planned Behavior Questionnaire. https://www.researchgate.net/publication/235913732_Constructing_a_Theory_of_Planned_Behavior_Questionnaire.

[40] Ajzen I. Constructing a TPB Questionnaire: Conceptual and Methodological Considerations. (2002): 2013. https://citeseerx.ist.psu.edu/viewdoc/download?doi=10.1.1.601.956&rep=rep1&type=pdf.

[41] Shen M., Mao Z., Yimin Z. (2010). Intervention strategies of Chinese Adult’s Exercise Behavior: The Integration of the TPB with the HAPA. China Sport Sci..

[42] Wilson P.M., Rogers W.T., Rogers W.M., Wild T.C. (2006). The psychological need satisfaction in exercise scale. J. Sport Exerc. Psychol..

[43] Barrett P. (2007). Structural equation modelling: Adjudging model fit. Personal. Individ. Differ..

[44] Bollen K.A., Stine R.A. (1992). Bootstrapping goodness-of-fit measures in structural equation models. Sociol. Methods Res..

[45] Lockwood C.M., MacKinnon D.P. Bootstrapping the standard error of the mediated effect. Proceedings of the 23rd Annual Meeting of SAS Users Group International.

[46] Schumacker R.E., Lomax R.G. (2004). A Beginner’s Guide to Structural Equation Modeling.

[47] Blue C.L. (1995). The predictive capacity of the theory of reasoned action and the theory of planned behavior in exercise research: An integrated literature review. Res. Nurs. Health.

[48] Hagger M.S., Chatzisarantis N.D.L., Biddle S. (2002). A meta-analytic review of the theories of reasoned action and planned behavior in physical activity: Predictive validity and the contribution of additional variables. J. Sport Exerc. Psychol..

[49] Hagger M.S., Chatzisarantis N.D.L., Culverhouse T., Biddle S. (2003). The processes by which perceived autonomy support in physical education promotes leisure-time physical activity intentions and behavior: A trans-contextual model. J. Educ. Psychol..

[50] Noar S.M., Zimmerman R.S. (2005). Health behavior theory and cumulative knowledge regarding health behaviors: Are we moving in the right direction?. Health Educ. Res..

[51] Beville J.M., Meyer M.R.U., Usdan S.L., Turner L.W., Jackson J.C., Lian B.E. (2014). Gender differences in college leisure time physical activity: Application of the theory of planned behavior and integrated behavioral model. J. Am. Coll. Health.

[52] Park S.-U., Lee C.G., Kim D.-K., Park J.-H., Jang D.-J. (2020). A Developmental Model for Predicting Sport Participation among Female Korean College Students. Int. J. Environ. Res. Public Health.

[53] McEachan R.R.C., Conner M., Taylor N.J., Lawton R.J. (2011). Prospective prediction of health-related behaviours with the theory of planned behaviour: A meta-analysis. Health Psychol. Rev..

[54] Godin G. (1993). The theories of reasoned action and planned behavior: Overview of findings, emerging research problems and usefulness for exercise promotion. J. Appl. Sport Psychol..

[55] Festinger L., Carlsmith J.M. (1959). Cognitive consequences of forced compliance. J. Abnorm. Soc. Psychol..

[56] Terry D.J., Hogg M.A. (1996). Group norms and the attitude-behavior relationship: A role for group identification. Personal. Soc. Psychol. Bull..

[57] Terry D.J., Hogg M.A., White K.M. (1999). The theory of planned behaviour: Self-identity, social identity and group norms. Br. J. Soc. Psychol..

